# Comparison of tumor biology of two distinct cell sub-populations in lung cancer stem cells

**DOI:** 10.18632/oncotarget.18451

**Published:** 2017-06-13

**Authors:** Jianyu Wang, Zhiwei Sun, Yongli Liu, Liangsheng Kong, Shixia Zhou, Junlin Tang, Hongmei Rosie Xing

**Affiliations:** ^1^ Laboratory of Translational Cancer Stem Cell Research, Institute of Life Sciences, Chong Qing Medical University, Chongqing, China

**Keywords:** cancer stem cells, symmetric division, asymmetric division, lung, BrdU

## Abstract

Characterization of the stem-like properties of cancer stem cells (CSCs) remain indirect and qualitative, especially the ability of CSCs to undergo asymmetric cell division for self renewal and differentiation, a unique property of cells of stem origin. It is partly due to the lack of stable cellular models of CSCs. In this study, we developed a new approach for CSC isolation and purification to derive a CSC-enriched cell line (LLC-SE). By conducting five consecutive rounds of single cell cloning using the LLC-SE cell line, we obtained two distinct sub-population of cells within the Lewis lung cancer CSCs that employed largely symmetric division for self-renewal (LLC-SD) or underwent asymmetric division for differentiation (LLC-ASD). LLC-SD and LLC-ASD cell lines could be stably passaged in culture and be distinguished by cell morphology, stem cell marker, spheroid formation and subcutaneous tumor initiation efficiency, as well as orthotopic lung tumor growth, progression and survival. The ability LLC-ASD cells to undergo asymmetric division was visualized and quantified by the asymmetric segregation of labeled BrdU and NUMB to one of the two daughter cells in anaphase cell division. The more stem-like LLC-SD cells exhibited higher capacity for tumorigenesis and progression and shorter survival. As few as 10 LLC-SD could initiate subcutaneous tumor growth when transplanted to the athymic mice. Collectively, these observations suggest that the SD-type of cells appear to be on the top of the hierarchical order of the CSCs. Furthermore, they have lead to generated cellular models of CSC self-renewal for future mechanistic investigations.

## INTRODUCTION

Lung cancer stem cells (CSCs) are a group of cells with self-renewal capability, which may play a key role in the initiation and development of lung cancer [1–3]. After the lung CSCs were identified and obtained through CD133 marker sorting, more recent research efforts have been put forward to understand how lung CSCs maintain their ability of self-renewal [1, 4–6]. However, some research indicated that the surfer marker could not distinguish the cancer stem-like cells [7]. In normal stem cells, the stemness is maintained by the symmetrical division(SD) for self-renewal and the asymmetric division(ASD) for undergoing differentiation [8, 9]. However, illustration of the ability of CSCs to undergo terminal differentiation is prevented by the constitutive activation of the growth signaling pathways [10–12]. Since ASD is a unique feature of normal stem cells, thus the stemness of CSCs can be probed through the demonstration of whether the CSCs can under go ASD.

Recent studies on human lung cancer cell A549 showed that a very small fraction (5%) of CSCs isolated from A549 could undergo ASD which was linked to the asymmetric segregation of BrdU-labeled parental DNA strands, as well as cell fate determinants, such as Numb, to only one of the two daughter cells [13–15]. However, no stable SD and ASD cell lines had been generated from lung CSCs derived from either A549 model or from lung cancer samples prior to this study. Thus, the role of SD and ASD in the initiation and development of lung cancer has not been explored.

In the present study, we report the cloning, identification and characterization of the SD and ASD cancer cells derived from mouse parental Lewis lung carcinoma cell line (LLC-Parental) which were named LLC-SD and LLC-ASD respectively. We show that LLC-SD and LLC-ASD CSCs could be stably maintained in culture and displayed distinct ability for tumor initiation and development. Whereas LLC-SD cells possessed long-lasting self-renewal and proliferative potential, as well as greater tumorigenicity and accelerated tumor progression compared with ASD cells. Half of ASD cells in cultures or in growing tumors displayed asymmetric segregation of both IdU labeled-DNA and cell-fate-determinant factor Numb as normal stem cells, and thus the ability to undergo ASD in a fashion that resembles normal stem cells.

## RESULTS

### Mouse Lewis lung cancer cell lines cultures contain a spheroid-forming subcomponent that exhibit stem cell properties

**Figure 1:**
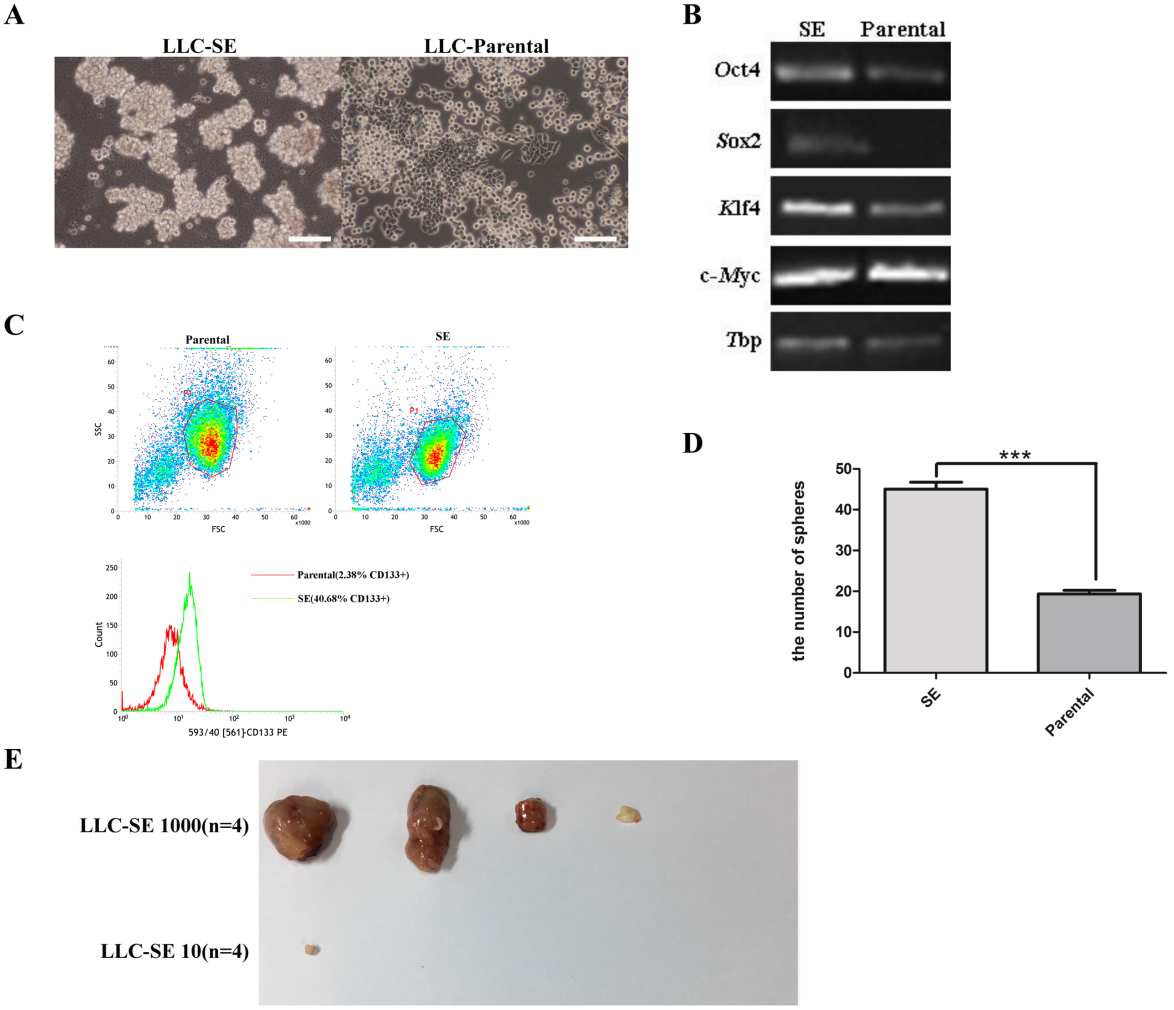
Mouse Lewis lung cancer cell line culture contains a spheroid-forming subcomponent **(A)** the morphology of spheroid forming cell lines from mouse Lewis lung cancer cell lines (LLC-SE) and LLC-Parental, bar=120um. **(B)** the analysis of mRNA expression of embryonic stem cell markers (*Oct4, Sox2, Klf4 and c-Myc*) in LLC-SE cell lines and LLC-Parental, the tata-Box binding protein gene (Tbp) was reference gene. **(C)** Flow cytometry of LLC-SE and LLC-Parental for CD133. **(D)** the analysis of the number of sphers in 6-well plate in one well of which 200 LLC-SE and LLC-Parental were seed, *P*=0.01. **(E)** Tumor formation in nude mice in which 10 and 1000 LLC-SE cells were injected respectively, n is the number of nude mice.

Eight rounds of selection for stable spheroid-forming floating cells resulted in the isolation of a highly comparable spheroid-enriched (SE) subcomponent from mouse Lewis lung cancer cells (LLC-Parental) (Figure 1A). The expression of normal stem-cell markers (*Oct4*, *Sox2*, *Klf4*, *c-Myc*) was markedly increased in the LLC-SE cells (Figure 1B). In addition, the expression of CD133, the most widely used cell surface marker for isolating lung tumor-initiating cells, known as cancer stem cells (CSCs), was also enhanced in LLC-SE cells (Figure 1C).

Compared with LLC-Parental lines, LLC-SE exhibited significantly augmented clonogenic spheroid formation efficiency (Figure 1D). Furthermore, the experiment of tumor growth in nude mice which is considered the gold standard for characterization of lung CSCs *in vivo* were carried out for LLC-SE and LLC-Parental. While subcutaneous tumor growth was observed with 1000 LLC-SE cells 3 weeks after tumor cell injection, no tumors were visible in mice injected with 1000 LLC-Parental cells (Figure 1E and Figure 4A). Moreover, as little as 1000 LLC-SE cells could initiate tumor growth in nude mice, the least amount of lung CSCs that exhibit tumorigenicity thus far reported.

**Figure 2:**
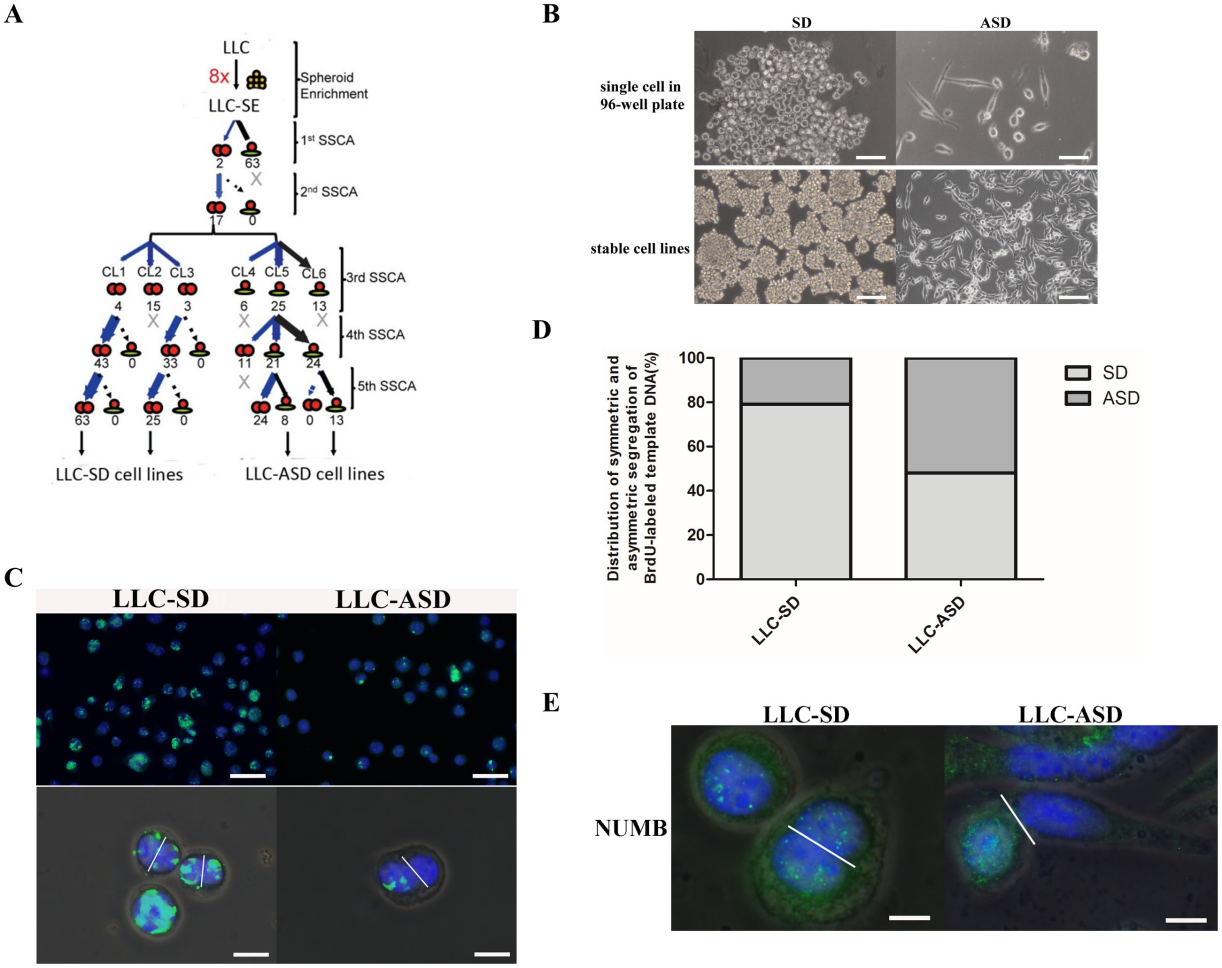
Cancer cells that have high clonogenic and cloning efficiency can undergo SD and ASD divisions **(A)** serial single cell cloning assay (SSCA) was set up as described in Methods. At each round, 180-wells were scored for monoclonal SD and ASD colony formation. **(B)** the morphology of single cell derived from LLC-SE in 96-well plate(top), bar=60um. And, the the morphology of stable symmetric division cell lines (LLC-SD) and asymmetric division cell lines (LLC-ASD) after 5 times SSCA of LLC-SE (bottom), bar=120um. **(C)** analysis of symmetric and asymmetric segregation of BrdU-labeled DNA during mitosis in LLC-SD and LLC-ASD. BrdU retention on day 7 after BrdU withdrawal in LLC-SD and LLC-ASD (top), bar=120um. Symmetric BrdU segregation between two daughter cells and asymmetric BrdU segregation between two daughter cells (bottom), bar=30um. **(D)** Quantification of SD and ASD cells in 100 dividing anaphase LLC-SD and LLC-ASD cells, respectively. **(E)** analysis of symmetric and asymmetric segregation of Numb in LLC-SD and LLC-ASD, bar=15um.

**Figure 3:**
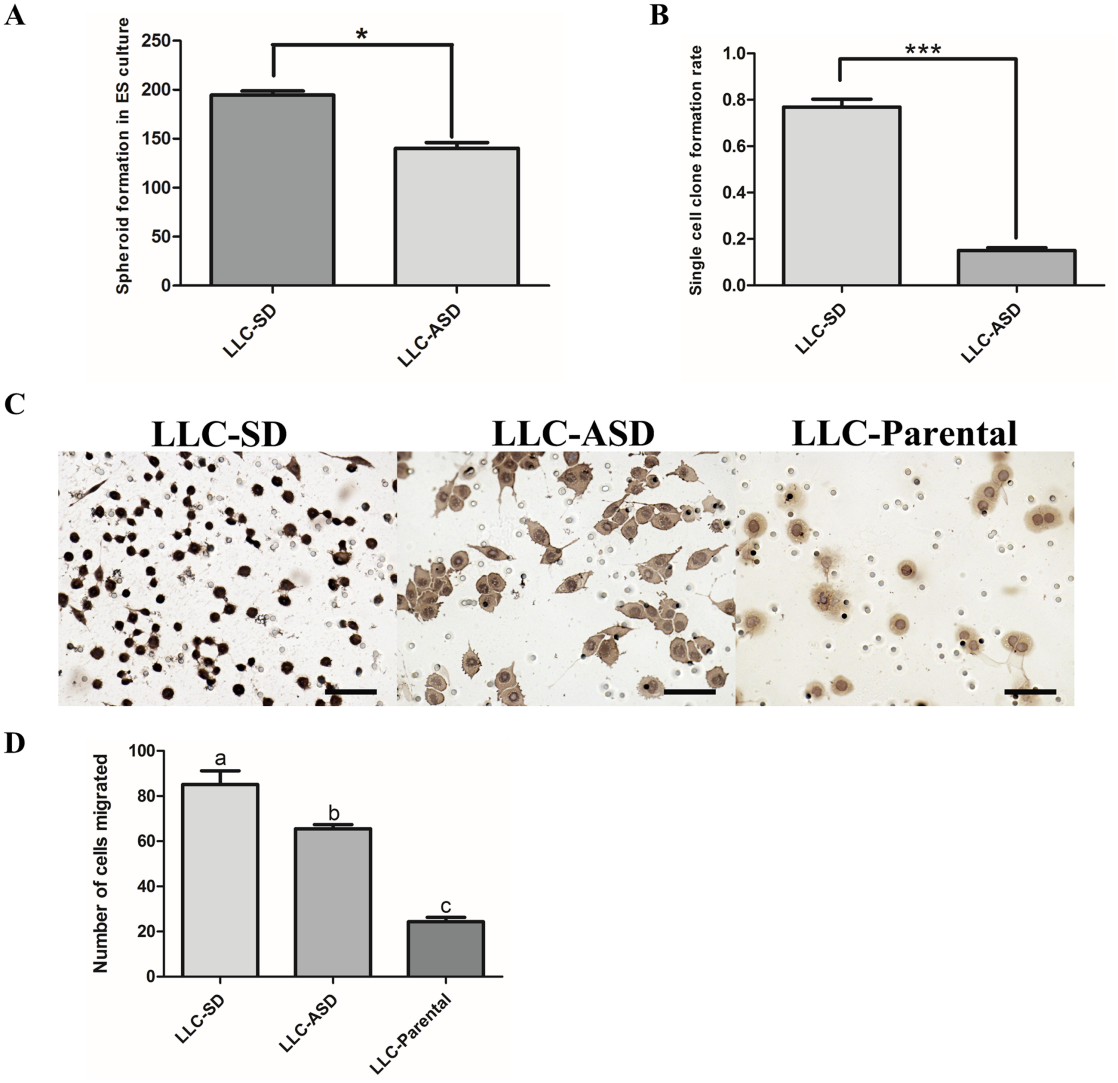
LLC-SD culture contains more stem-like cancer cells **(A)** the analysis of the sphere formation in FBS-free medium in which 200 LLC-SD and LLC-ASD cells were seeded respectively, *: p<0.05. **(B)** the analysis of single cell cloning formation in which 180 wells were assaied, ***: p<0.01. **(C-D)** LLC-SD exhibited enhanced migration compared with ASD cell lines, bar=120um.

**Figure 4:**
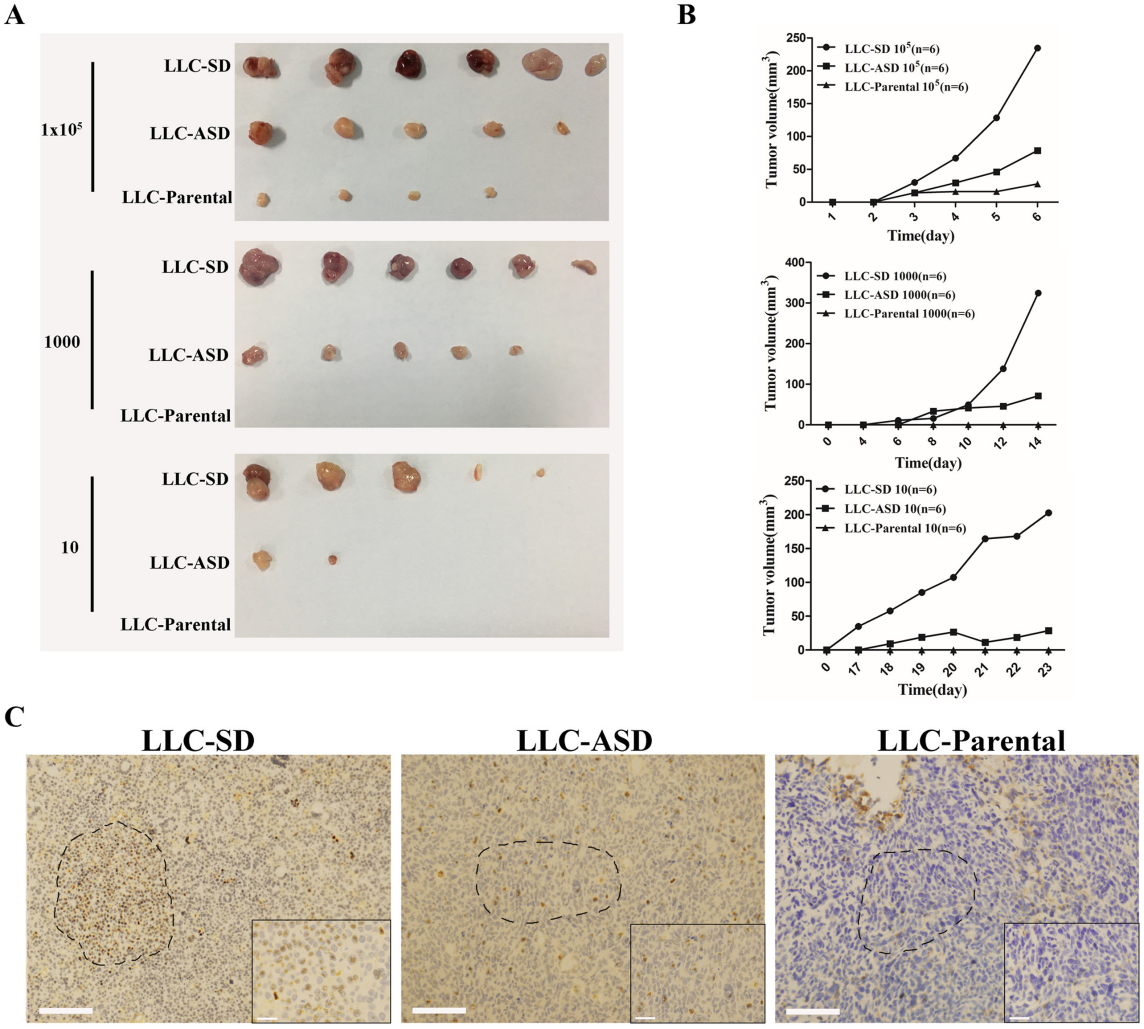
Tumorigenicity of LLC-SD and LLC-ASD in nude mice **(A)** the tumor formation in nude mice to which 10^5^, 1000 and 10 LLC-SD, LLC-ASD and LLC-Parental cells were injected respectively. **(B)** the tumor volume tracking curve of 10^5^, 1000 and 10 LLC-SD, LLC-ASD and LLC-Parental cells respectively. **(C)** immunohistochemistry analysis of BrdU-positive cells in growing tumors derived from 10^5^ LLC-SD, LLC-ASD and LLC-Parental cells, bar=285um. the dotted line indicates the enlarged area, bar=30um.

### LLC-SE that have high clonogenic and cloning efficiency can undergo SD and ASD divisions

In the experiment of tumor growth in nude mice, injection of 10 LLC-SE cells failed to initiate tumor growth 4 weeks after injection (Figure 1E). Careful observation of the LLC-SE revealed the presence of distinct cell types that could be distinguished by size and morphology of the clones, indicating that although LLC-SE cell culture were enriched with cells that possess characteristics of the lung CSCs, it may contain non authentic CSCs.

In order to further verify whether there were different cell types in LLC-SE, the single cell cloning assay in 96-well plate was performed which is the widely used assay in stem cell research to assess the ability of stem cell self renewal. We conducted a total of five successive rounds of single-cell cloning assay for selecting individual cells within the SE-component that exhibit high cloning efficiency (Figure 2A). Using this assay, two types of cells were obtained which shared the unique morphological features of normal stem cells undergoing symmetrical division (SD) or asymmetric division(ASD). Both cell types could be expanded and passaged stably to yield two derivative cell lines: the LLC-SD and LLC-ASD, respectively (Figure 2B). The ASD colonies typically consisted of roughly 50% large spindle shape attached cells and the other 50% loosely attached small round cells, whereas SD colonies consisted of exclusively small round cells that were morphologically undifferentiated (Figure 2B).

At the molecular level, SD and ASD divisions in normal stem cells can be distinguished by patterns of chromosome segregation, as well as the patterns of partitioning of cell fate determinants such as Numb in mitoses. We labeled nuclear DNA of LLC-SD and LLC-ASD cells with the BrdU. On day 7 after BrdU withdrawal, LLC-SD cultures were found to contain more and brighter BrdU-positive cells than the LLC-ASD cultures (Figure 2C). Among 100 anaphase LLC-SD cells analyzed, symmetric partition of BrdU-labeled DNA templates occurred in 79 cells, while the other 21 daughter cell pairs had asymmetric BrdU segregation (Figure 2D). In contrast, more asymmetric BrdU segregation was observed in anaphase LLC-ASD cells (asymmetric/symmetric=51/49) (Figure 2D). The ability of over 50% of LLC-ASD cells in culture to undergo asymmetric division was confirmed by the asymmetric segregation of the stem cell fate determinant Numb in the two daughter cells, whereas LLC-SD culture exhibited mostly symmetric Numb distribution (Figure 2E). Taken together, monoclonal enrichment didn’t alter the ability of both LLC-SD and LLC-ASD cells to undergo asymmetric divisions, a property that is only present in cells of stem origin. Nevertheless, the fraction of cells that underwent ASD in the LLC-ASD culture was higher than that of in the LLC-SD culture. ASD could be distinguished by their unique morphology, as well as by molecular properties of asymmetric segregation of both BrdU labeled-DNA and the cell-fate-determinant factor Numb.

### LLC-SD culture contain more stem-like cells

The difference between the LLC-SD and LLC-ASD in stem cell self-renewal *in vitro* was assessed by the clonogenic-spheroid formation assay (Methods, Figure 3A) and by the single cell cloning formation assay (Figure 3B). LLC-SD cell culture displayed significantly higher self-renewal ability and clonogenic activities, respectively. The metastatic potential was measured using a modified Matrigel invasion assay (Methods) in which LLC-SD cells exhibited greater *in vitro* invasion than the LLC-ASD and LLC-Parental cells (Figure 3C and 3D). These results collectively indicated that LLC-SD culture contained more stem-like CSCs than the LLC-ASD culture *in vitro*.

### *In vivo* characterization of LLC-SD and LLC-ASD

*In vivo*, we first compared the differences in tumorigenic capability of LLC-SD and ASD cells using the nude mice subcutaneous tumor cell transplantation model. Athymic nude mice were injected with the following three doses of cells for each cell line, respectively:10^5^, 1000 and 10 cells per mouse. While the LLC-SD were most efficient in tumor initiation in all 3 cell doses tested, the LLC-Parental cells were the least tumorigenic, as evident by the sizes of the tumors (Figure 4A) and the growth kinetics of the tumors (Figure 4B).

Prior to this study, the presence of lung CSCs in the growing xenograft tumors have not been identified and visualized. 10^5^ LLC-SD, LLC-ASD and LLC-Parental cells, labeled with BrdU, were injected subcutaneously to the nude mice. LLC-SD tumors retained the most BrdU-positive cells after 2 weeks of injection. In contrast, the presence of BrdU-positive cells in LLC-ASD tumors was sparse, and there were almost no BrdU-positive cells in LLC-Parental tumors (Figure 4C). Most importantly, BrdU-positive slow cycling cells in the growing tumors can undergo SD and ASD cell divisions (Figure 4C). This was the first *in vivo* evidence that BrdU-retaining CSCs could undergo SD and ASD in the growing tumor, indicating that CSC self-renewal and differentiation *in vivo* can be visualized by BrdU labeling and immunostainging.

**Figure 5:**
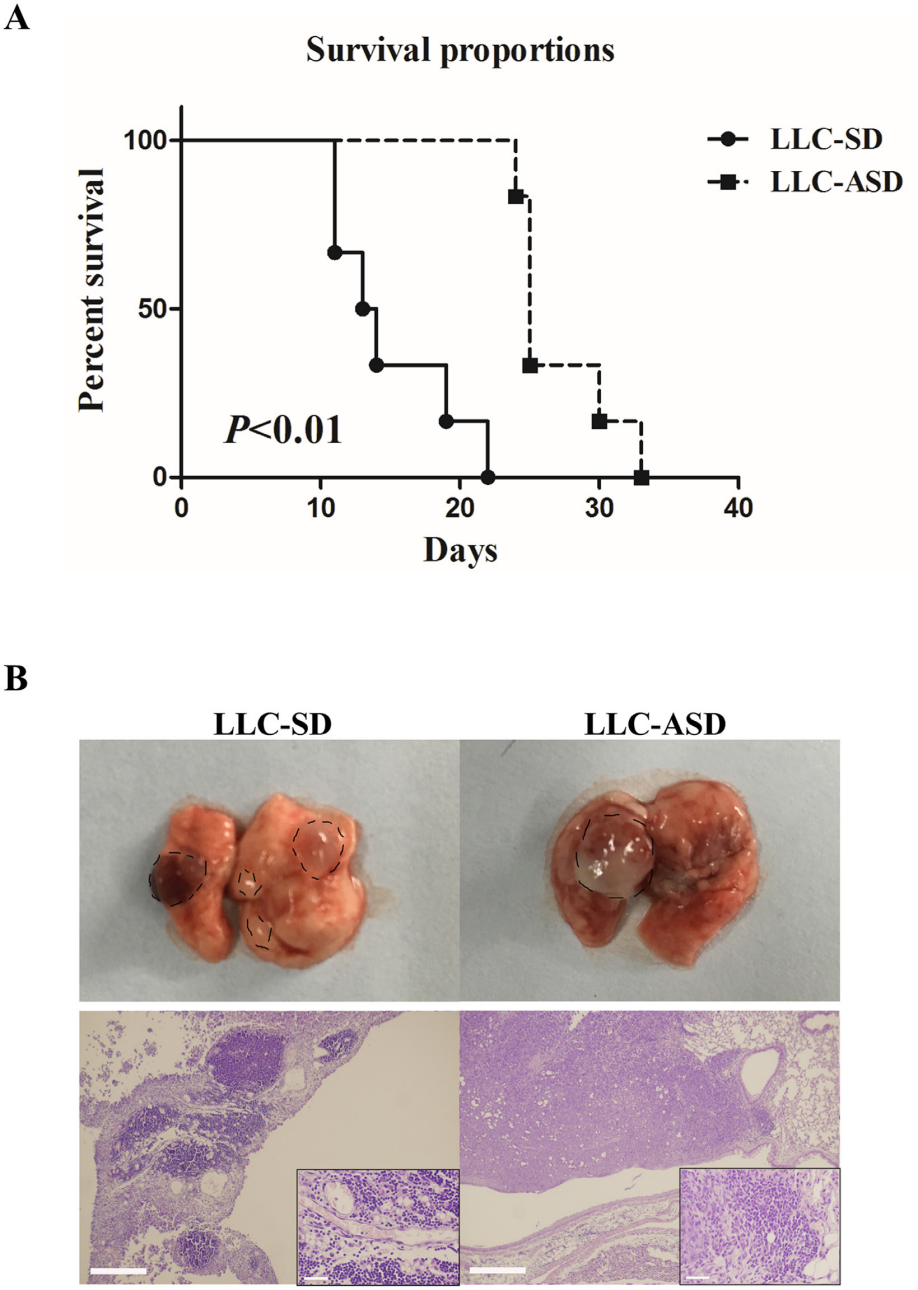
Cancer biology of LLC-SD and LLC-ASD in C57BL/6 mice **(A)** the survival proportion of C57/B6 mice injected with 10^5^ LLC-SD and 10^5^ LLC-ASD cells respectively. **(B)** immunohistochemistry analysis of the tumor derived from LLC-SD and LLC-ASD in C57/B6 mice lung *in situ*.

Prior to this study, the role of SD and ASD CSCs in tumorigenesis has not been characterized due to the lack of syngeneic animal model of tumorigenesis. We have developed a clinically relevant syngeneic LLC orthotopic mouse model of lung cancer which allowed the evaluation of the tumor biology characteristics of LLC-SD and LLC-ASD in C57/B6 mice. LLC-SD and LLC-ASD cells were injected in the left lung in the syngeneic intact C57/B6 mice. Survival assay was performed in mice orthotopically transplanted with 10^5^ cells of LLC-SD and LLC-ASD. While the mice injected with LLC-SD died from 11h days until the 22th day, the mice injected with LLC-ASD died from 24th days until the 33th day after injection (Figure 5A). Thus, the survival curve of LLC-ASD was significantly right-shifted (Figure 5A). An additional two sets of mice orthotopically transplanted with 10^5^ LLC-SD and LLC-ASD cells, respectively were monitored more tumor progression within the thoracic cavity. All mice were sacrificed on day 14 post tumor cell transplantation. At the site of tumor cell transplantation, orthotopic tumors were developed in 6/6 mice injected with LLC-SD cells, and in 4/6 mice injected with LLC-ASD. Visible metastatic foci were found at mediastinal lymph nodes of 4/6 mice and at the right lung of 3/6 mice injected with LLC-SD cells, respectively. In contrast, lymph node and right lung metastases were observed in only 2/6 and 0/6 mice injected with LLC-ASD cells, respectively (Table 1). LLC-SD and LLC-ASD tumors *in situ* (left lung) were analyzed by H&E staining. The invasiveness of LLC-SD cells was evident by the presence of LLC-SD cells in the blood vessels (Figure 5B). The more pronounced *in vivo* metastatic potential of LLC-SD cells (Figure 5B and Table 1) was consistent with the *in vitro* invasive behavior determined by the modified Matrigel invasion assay (Figure 3C and 3D).

**Table 1:** Characterization of orthotopic LLC-SD and LLC-ASD tumorigenesis and metastatic progression

	Orthotopic site	Metastasis	
	Lung (left)	Mediastinal lymph	Lung(right)
LLC-SD 10^5^ cells (n=6)	6/6	5/6	3/6
LLC-ASD 10^5^ cells(n=6)	4/6	2/6	0/6

### SD and ASD cell lines could be obtained from human breast cancer cells MDA-MB-435

**Figure 6:**
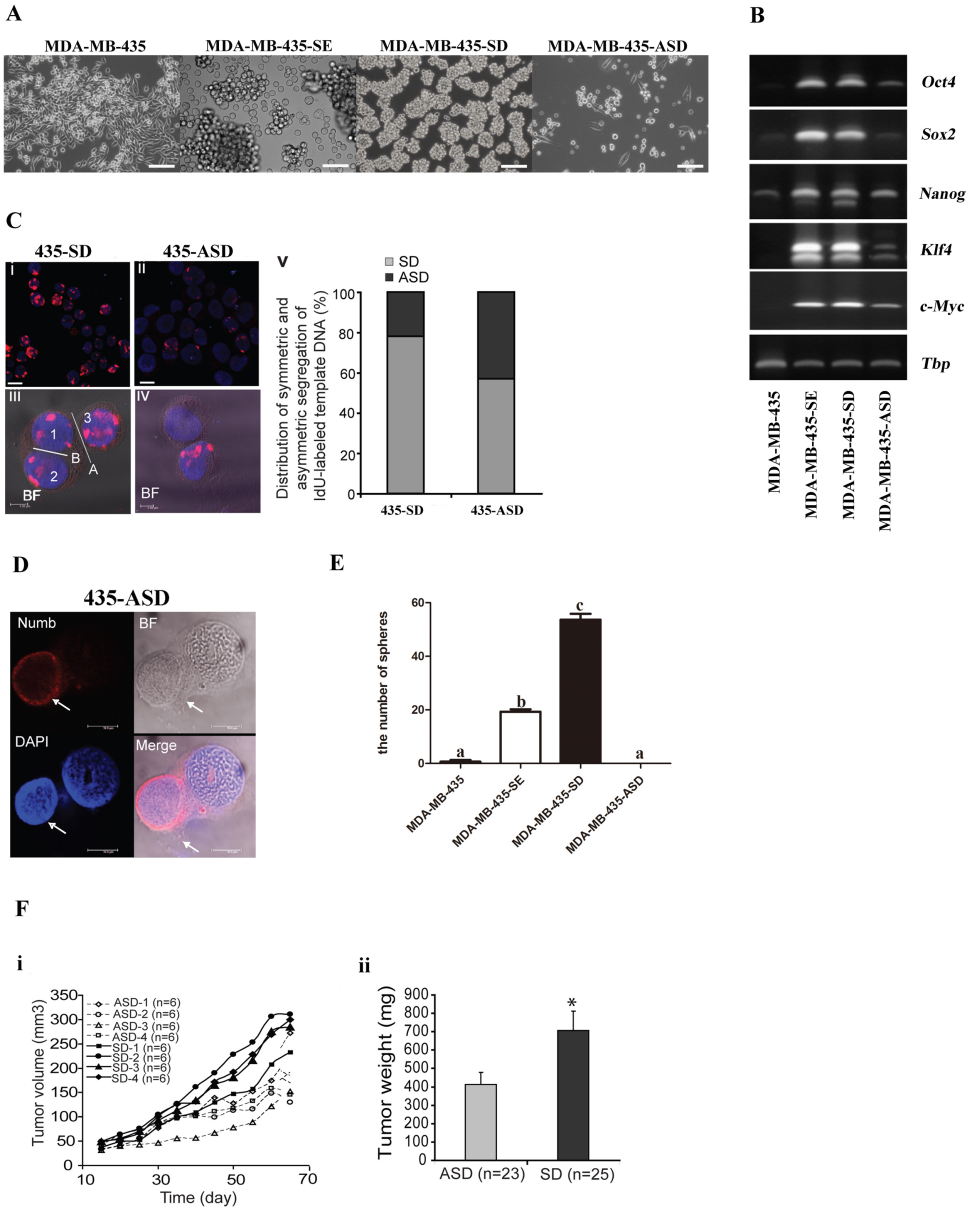
SD and ASD cell lines could be obtained from human cancer cells MDA-MB-435 **(A)** the morphology of MDA-MB-435, MDA-MB-435-SE, MDA-MB-435-SD and MDA-MB-435-ASD, bar=120um. **(B)** the analysis of mRNA expression of embryonic stem cell markers (Oct4, Sox2, Nanog, Klf4 and c-Myc) in MDA-MB-435, MDA-MB-435-SE, MDA-MB-435-SD and MDA-MB-435-ASD, the tata-Box binding protein gene (Tbp) was reference gene. **(C)** Analysis of symmetric and asymmetric segregation of BrdU-labeled DNA during mitosis in MDA-MB-435-SD and MDA-MB-435-ASD. BrdU retention on day 6 after BrdU withdrawal in MDA-MB-435-SD (i) and MDA-MB-435-ASD (ii). (iii), #1 and #2: symmetric BrdU segregation between two daughter cells. #3: an adjacent BrdU-positive cell. (iv), asymmetric BrdU segregation between two daughter cells. (v) Quantification of SD and ASD cells in 100 dividing anaphase MDA-MB-435-SD and MDA-MB-435-ASD cells, respectively. Scale bars: 10 uM in (i-ii), 5 uM in (iii-iv). **(D)** asymmetric partitioning of Numb, in a pair of newly formed daughter cells in a dividing anaphase MDA-MB-435-ASD cell. **(E)** MDA-MB-435-SD exhibited significantly enhanced clonogeneicity and sphere-forming capability compared with corresponding MDA-MB-435, MDA-MB-435-SE and MDA-MB-435-ASD. a, b and c: different letters represent a significant difference, p < 0.05. **(F)** Tumor growth of four distinctive MDA-MB-435-SD and MDA-MB-435-ASD cell lines. (i), tumor volume; (ii), tumor weight. *: p=0.0025 determined by one-tailed t-test.

To access the feasibility of establishing SD- and ASD-cell lines from human cancer cell lines using the simple and novel approach we have developed, the human cancer cell lines MDA-MB-435 was used. We successfully obtained the stable SE, SD and ASD MDA-MB-435-derivative cell lines: MDA-MB-435-SE, MDA-MB-435-SD and MDA-MB-435-ASD (Figure 6A). The expression of normal stem-cell markers *(Oct4*, *Sox2*, *Nanog*, *Klf4* amd *c-Myc*) was markedly increased in the MDA-MB-435-SE, MDA-MB-435-SD and MDA-MB-435-ASD than the parental MDA-MB-435 cells, and the increase was more pronounced in MDA-MB-435-SE and the MDA-MB-435-SD cells (Figure 6B).

BrdU-labeling experiment was conduct for quantitative analysis of symmetric and asymmetrical chromosome segregation. Symmetric partition of BrdU-labeled DNA templates occurred in 78 cells, while the other 22 daughter cell pairs had asymmetric BrdU segregation (Figure 6C). In contrast, more asymmetric BrdU segregation was observed in anaphase MDA-MB-435-ASD cells (asymmetric/symmetric=58/42) (Figure 6C). Nevertheless, the SD- and ASD-cells didn’t loose the ability to undergo asymmetric and symmetric division, respectively. The asymmetric segregation of the Numb in the two daughter cells was also observed in the MDA-MB-435-ASD (Figure 6D).

*In vitro*, MDA-MB-435-SD cells exhibited significantly augmented clonogenic spheroid formation efficiency (58/100). MDA-MB-435-SE had less number of spheroids (20/100) compared with MDA-MB-435-SD cells and with MDA-MB-435 cells (2/100). No spheroids were obtained from MDA-MB-435-ASD (0/100) cells (Figure 6E). We compared the differences in tumorigenic capability of MDA-MB-435-SD and MDA-MB-435-ASD cells using the nude mice subcutaneous tumor cell transplantation model in which 1000 cells were injected. MDA-MB-435-SD had faster tumor growth rate than the MDA-MB-435-ASD (Figure 6D–6i). MDA-435-SD tumors weighed heavier than the MDA-MB-435-ASD tumors (Figure 6D–6ii).

In summary, we show that there is a small fraction of cancer cells within LLC that possess normal stem cell-like properties. Most importantly, they can undergo stem-cell like symmetric and asymmetric division, both *in vitro* and *in vivo*. We also show that monoclonal derived SD and ASD cells have markedly different properties of clonogenic and cloning efficiencies *in vitro* and tumor biology properties *in vivo*. Furthermore, the SD and ASD cell lines established from human breast cancer cell line MDA-MB-435 exhibited similar differences in stem-like properties both *in vitro* and *in vivo*, attesting the validity of the new approach we have developed for the isolation, purification, cloning, expansion and characterization of SD- and ASD-subpopulations of cells.

## DISCUSSION

While symmetric division (SD) and asymmetric division (ASD) were an evolutionarily-conserved development feature of germ line or stem cells [10, 16–18], experimental evidence for the presence of ASD-type cell division in cancer cell lines, prior to this study, was limited and qualitative [19]. The isolation and cloning of SD and ASD cancer cells in Lewis lung cancer cells (LLC) (Figure 2A and 2B), followed by quantification and molecular characterization of SD and ASD divisions both *in vitro* (Figure 2C and 2D) and *in vivo* (Figure 4C) have collectively demonstrated that cancer cell lines may contain both SD and ASD types of stem-like cancer cells. The approach presented here can be applied to isolate SD and ASD-type of CSCs from other human cancer cell lines as exemplified by the human breast cancer cell line MDA-MB-435. We are undertaking studies to optimize the isolation of SD and ASD cells from other human cancer cell lines and from freshly resected tumor samples.

The Numb-Notch pathway is the best-characterized molecular mechanism underlying normal stem cell ASD division. Its asymmetric partitioning is linked to the asymmetric fate of the two daughter cells [14, 15, 20, 21]. In our study, in addition to asymmetric partitioning of BrdU-labeled DNA to the two daughter cells of an ASD division, we also observed the asymmetric segregation of Numb to the less differentiated daughter cell (Figure 2E). Future studies will examine the role of this pathway in regulating ASD division and in cell fate specification in lung CSCs.

Our observed high proportion of ASD cells in both the monoclonal SD (21%) and ASD (51%) cultures (Figure 2C and 2D) is in contrast with the reported low occurrence of ASD division [19, 22, 23]. The presence of higher content of cells that can undergo ASD is the results of purification, monoclonal cloning and expansion. The SD and ASD phenotypes in our models are stable since cells within a given monoclonal SD or ASD colony, chosen at random, maintained the ability to divide via both SD and ASD, respectively, in the subsequent rounds of cloning (Figure 2B). More importantly, we show for the first time that SD and ASD divisions could occur in growing tumors, supported by the evidence of symmetric and asymmetric BrdU-segregation of parental DNA in fresh harvested LLC-SD and LLC-ASD tumors (Figure 4C).

In the literature report, Lung CSCs were obtained by marker sorting or suspension sphere enriched [1, 6, 24, 25]. In the first part of this study, the spheroid enriched cells from LLC (LLC-SE) were obtained which were verified to be characteristic of lung CSCs. Only 1000 LLC-SE cells initiated tumor growth in nude mice (Figure 1). However, according to our study, there were at least two distinct cell types in LLC-SE, namely LLC-SD and LLC-ASD both of which were more stem-like and more tumorigenic than LLC-SE (Figure 4A). These observations indicate that the so called “lung CSCs” in the literature report obtained via spheroid enrichment were not a pure CSC population. This study demonstrated for the first time that the CSCs hierarchy was ordered in such that LLC-SD was on the top, LLC-ASD was in the middle and the LLC-Parental in the bottom, with respect to stemness properties [26].

Cancer stem cell therapy may hold promises to more effective treatment for cancer [2, 27, 28]. However, before CSC-based therapies can be developed, we have to first understand how CSCs self-renewal is regulated and the differences between the stem cell renewal under normal development and under malignancy. While the ability of marker sorted or spheroid enriched CSCs to undergo asymmetric division was reported in the recent literature, the illustration was qualitative [22, 23]. Prior to this study, in-depth assessment of quantitative differences in stemness *in vitro* and tumor biology properties *in vivo* are prohibitive due to the lack of stable cellular models of SD and ASD CSCs, as well as as well as syngeneic animal models for tumorigenesis. Although orthotopic implantation of freshly harvested tumor tissues would give arise to more robust spontaneous metastases [29, 30], freshly harvested tumor tissues contain heterogeneous cell populations including a small portion of SD- and the ASD cells, as well as majority of more differentiated cells that do not exhibit stem-cell like properties. For this consideration, orthotopic transplantation of purified SD and ASD cell lines was performed so that the differences observed can be ascribed to the differences in stem-like characteristics between the SD and the ASD cells. Further, labelling SD and ASD cells with different colors of fluorescent proteins will allow real-time co-imaging both *in vitro* and *in vivo*. It will be of great interest to visualize the interactions between the two CSC cell types and the importance of such interactions for cancer initiation, progression and dissemination [31–33]. Investigations are undergoing to illustrate the mechanisms that regulate symmetric division and asymmetric division in cancer stem cells using the cellular and animal models generated and characterized in the study presented here.

## MATERIALS AND METHODS

### Animals

All mice used in the study were obtained from the core facility of Experimental Animal Centre in Chongqing Medical University. All animal work was conducted in accordance with an approved protocol and carried out in accordance with the institutional animal welfare guidelines of the Chongqing Medical University.

### Cell culture

Mouse parental Lewis lung carcinoma cell line (LLC-Parental) and MDA-MB-435 were kindly provided by Dr. Robert Hoffman (University of California San Diego). LLC-Parental and MDA-MB-435 were maintained in DMEM high glucose supplemented (Gibico) with 10% fetal bovine serum (FBS) (ExCell Bio). Spheroid-enriched (SE) cell lines generated from above LLC-Parental (LLC-SE) and MDA-MB-435 were maintained in DMEM high glucose supplemented with 10% FBS. The monoclonal ASD cell lines generated from LLC-SE cell lines (LLC-ASD) and MDA-MB-435 were maintained in DMEM high glucose supplemented with 5% FBS. The monoclonal SD cell lines generated from LLC-SE cell lines (LLC-SD) and MDA-MB-435 were maintained in DMEM/F12-based normal stem-cell media (Gibico), supplemented with 20ng/ml EGF (BD), 20ng/ml FGF (BD), 2% B27 (BD) and 1% PS (Hyclone).

### Enrichment of the spheroid-forming component (SE) from monolayer parental cancer cell-line cultures

The two key elements in our enrichment approach were the use of attachment culture condition and repetitive selection for cells that consistently display anchorage-independent spheroid growth. Monolayer cultures were let grow to 90% confluence, at which time floating single cells or spheroids were collected, re-suspended, re-plated immediately and grown until reaching sub-confluence. By repeating this procedure eight consecutive times, the resulting cultures were enriched in spheroid-forming cells that remained afloat in attachment culture condition. We termed such stem-like cell lines “SE-enriched”.

### Serial single cloning assay (SSCA)

The SSCA enabled the isolation of individual single cancer cell that exhibited long lasting proliferative potential. To set up this assay, a single-cell suspension of SE was plated at 1cell/well in 96-well plates. Single-cell plating was confirmed by microscopic examination and wells containing more than one cells were marked. After two weeks of growth, SD and ASD colonies were counted under microscopy based on their respective characteristic morphology. Then one SD and one ASD colony from each cell line were randomly chosen for immediately setting up of the second round of cloning. This cloning procedure was applied for up to a total of five rounds. At the end of the 5th round of SSCA (or the last round in cell lines that could not complete five rounds of SSCA), 4-6 SD and ASD colonies were randomly selected and seeded into 24-well plates for expansion and establishment of single-cell-derived monoclonal SD and ASD cell lines.

### Clonogenic spheroid formation assay (CSFA)

In the CSFA, each spheroid was produced by clonogenic expansion of a cancer cell that had both high clonogenicity and stem-like spheroid forming ability. To set up this assay, single-cell suspensions were mixed with 0.1% agar (Sigma) (to prevent cell aggregation) and plated at 200 cells/well in 6-well plates. Two ml fresh media were added on the top of the agar layer. After two weeks in culture, clonogenic spheroids that consisting of a minimum of 50 cells were counted under microscopy.

### Single cell cloning assay

The single-cell suspensions were prepared at the concentration of 10 cells/ml using gradient dilution. 100μl diluted suspensions were added to every well in 96-well plates. Single-cell plating was confirmed by microscopic examination and wells containing more than one cell were marked. After 10 days of incubation, colonies that consisting of a minimum of 50 cells were counted under microscopy.

### Transwell migration assay

The monoclonal SD and ASD cells were re-suspended in fully supplemented medium and were then seeded at 2,000 cells per well for migration assay into transwell inserts (8 μm pore size, BD Falcon). 10%FBS was used as chemo-attractant in the lower chamber. After 24 hours of incubation, cells were removed from the upper surface of the porus membrane with a cotton swab, followed by fixation of cells migrated to the lower surface with 70% ethanol for 1 hour and staining with hematoxylin for 15 minutes. Stained cells were counted under light microscopy and 10 random fields from three replicate transwells were counted. The number of cells that had migrated was presented as number of cells counted per field of the porous membrane.

### BrdU-labeling, analysis of symmetric and asymmetrical chromosome segregation

Labeling of DNA templates was achieved by the addition of 10μM BrdU (Sigma) to the culture medium for 7 days followed by BrdU withdrawal. BrdU immunostaining was performed as described below. Anaphase cells were identified if both sets of chromosomes were distinctly separated and had parallel and condensed chromatin. Single anaphase cells prior to cytokinesis were verified by phase contrast microscopy. An BrdU-positive anaphase cell was determined to have asymmetric partitioning of BrdU-labeled DNA if one daughter cell contains a set of chromosomes that is strongly BrdU-positive while the other daughter cell is completely or near completely BrdU-negative. In contrast, if symmetric segregation of BrdU-labeled DNA occurs, the two daughter cells both contain BrdU-labeled DNAs. A total of 100 SD and 100 ASD cells at anaphase were analyzed on day 6 after BrdU withdrawal. Images of the BrdU-labeling analysis were taken with Olympus FSX100 Box-Type Fluorescence Imaging Device.

### Orthotopic tumor transplantation studies in C57BL/6 mice

To set up the *in vivo* experiments, The single-cell suspensions were enriched to the concentration of 10^5^ cells/ml and mixed with equivalent volume of Growth Factor Reduced Matrigel Matrix (Corning). 20μl cell suspensions containing Matrigel Matrix were injected orthotopically into the left lobe of the lungs of C57BL/6 mice all of which were female and 6-8-week old using 1 ml syringe. For the survival experiments, the time of death of every mouse was recorded after orthotopic tumor transplantation. For the experiments of tumorigenesis, the mice were dissected on day 14 to determine the tumors *in situ* and the extent of thoracic metastasis.

### Tumor transplantation studies in BALB/c nude mice

The single-cell suspensions of specific concentrations were mixed with equivalent volume of Growth Factor Reduced Matrigel Matrix as described above. 100μl suspensions containing Matrigel Matrix were injected subcutaneously into both insides of the hind legs of each mouse all of which were male and 6-8 weeks old. Tumor growth was measured every two days, and tumor volume was calculated as V= (length x width^2^)/2.

### Immunofluorescence and immunohistochemistry staining

For immunohistochemistry, 5 micron-cut paraffin-embedded tumor sections were deparaffinized, hydrated and H&E stained. For immunofluorescence staining, cells cultured on chamber slides or 5 micron-cut tumor sections were fixed with formalin (1:10 dilution), incubated with blocking solution (10% goat serum in 0.1% Triton X-100 PBS) for 1 hour and followed by incubation with the primary antibody (anti-BrdU, Millipore) diluted 1:200 in blocking solutions at 4°C overnight. After washing, slides were incubated with the fluorophore-labeled secondary antibody (Proteintech) diluted 1:2000 in blocking solutions for 30 min at room temperature. The slides were then mounted in Vectashield mounting medium with 4’,6-diamidino-2-phenyl-indole (DAPI), and images were captured and analyzed using the Olympus FSX100 Box-Type Fluorescence Imaging Device. The NUMB immunofluorescence were similar with the BrdU without Triton in which the primary abtibody was obtained from Millipore and the secondary antibody was obtained from the Proteintech.

### Flow cytometry

0.5-1 million cells of each single-cell suspension was incubated with the respective conjugated antibody for 15 min at 4°C and analyzed with a BD LSR. Unstained cells were included as negative controls for each FACS analysis. The antibodies used in this study were: anti-CD133-PE (eBioscience). The data were analyzed using BD FACS software.

### Reverse transcription polymerase chain reaction (RT-PCR)

Total RNA was extracted used by TRIZOL (Invitrogen) according to the manufacturer’s protocol. Standard RT-PCR was conducted using PrimeScript RT Master Mix (Takara) according to the manufacturer’s instructions. The sequences of PCR primers are listed in Table 2.

**Table 2:** PCR primer sequence

Gene name	Forward primers	Reverse primers
mouse *Oct4*	TTCCCTCTGTTCCCGTCACT	TGTCTACCTCCCTTGCCTTG
mouse *Sox2*	AGGGCTGGGAGAAAGAAGAG	ATCTGGCGGAGAATAGTTGG
mouse *Klf4*	GGACCACCTTGCCTTACACA	GACTTGCTGGGAACTTGACC
mouse *c-Myc*	ACAAGAGGCGGACACACAAC	TTGAATGGACAGGATGTAGGC
mouse *Tbp*	GCGACCCTCACATCAAACT	CAGTGCCACATACCAACT
human *Oct4*	CGAAAGAGAAAGCGAACCAG	TGAAGTGAGGGCTCCCATAG
human *Sox2*	CACAACTCGGAGATCAGCAA	GTTCATGTGCGCGTAACTGT
human *Nanog*	AGATGCCTCACACGGAGACT	TCTGGAACCAGGTCTTCACC
human *Klf4*	TTCTCTCCAATTCGCTGACC	GTACACCGGGTCCAATTCTG
human *c-Myc*	TTCGGGTAGTGGAAAACCAG	CAGCAGCTCGAATTTCTTCC
human *Tbp*	TATAATCCCAAGCGGTTTGC	CACAGCTCCCCACCATATTC

### Statistical analysis

All data were presented as mean ± standard deviation. Tumor volumes were analyzed using an ANOVA when data satisfied the Gaussian distribution assessed by the D'Agostino-Pearson normality test and the F test for equal variance. When two groups were compared, the Student’s t-test with Welch’s correction was used. By default, two-tailed tests were performed. KaplanMeier’s method was used for survival analysis. P <0.05 was considered significant statistically and is marked with an asterisk. P < 0.01 was considered highly significant statistically and is marked with adouble asterisk. All statistical analyses were performed using Graphpad Prism version 5.0.

## Abbreviations

CSCs: cancer stem cells
SD: symmetric division
ASD: asymmetric division
LLC-Parental: mouse parental Lewis lung carcinoma cell line
SE: spheroid-enriched
SSCA: Serial single cloning assay
CSFA: Clonogenic spheroid formation assay
DAPI: 4’,6-diamidino-2-phenyl-indole
